# Supplementary material for: Evidence supported by Mendelian randomization: impact on inflammatory factors in knee osteoarthritis
Source: Front Med (Lausanne). 2024 May 28;11:1382836. doi: 10.3389/fmed.2024.1382836 (PMC11165061; doi:10.3389/fmed.2024.1382836)
Supplement: Supplementary file 1 [file Data_Sheet_1.ZIP › data/GCST90274800_5e6/800.leaveoneout.pdf]

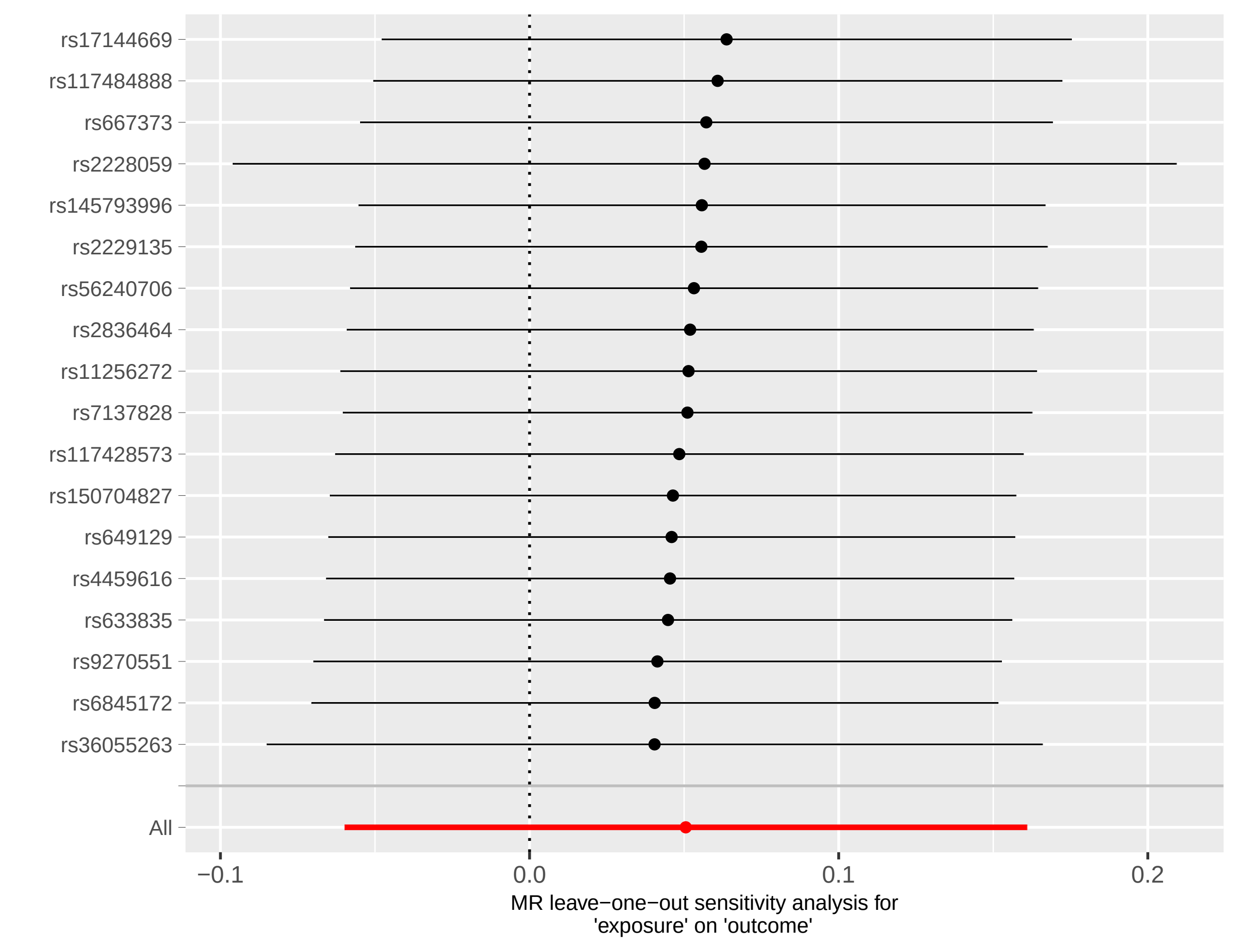

rs17144669
rs117484888
rs667373
rs2228059
rs145793996
rs2229135
rs56240706
rs2836464
rs11256272
rs7137828
rs117428573
rs150704827
rs649129
rs4459616
rs633835
rs9270551
rs6845172
rs36055263
All
−0.1
0.0
0.1
0.2
MR leave−one−out sensitivity analysis for
'exposure' on 'outcome'
